# The correlation between novel peripheral blood cell ratios and 90-day mortality in patients with acute ischemic stroke

**DOI:** 10.1371/journal.pone.0238312

**Published:** 2020-08-28

**Authors:** Xiaofeng Cao, Qing Zhu, Xin Xia, Beibei Yao, Seng Liang, Zhaoyao Chen, Minghua Wu

**Affiliations:** 1 Affiliated Hospital of Nanjing University of Chinese Medicine, Nanjing, China; 2 Department of Neurology, Jiangsu Province Hospital of Chinese Medicine, Nanjing, China; 3 The First Clinical Medical College, Nanjing University of Chinese Medicine, Nanjing, China; Hospital Dr. Rafael A. Calderón Guardia, CCSS, COSTA RICA

## Abstract

**Background:**

We aimed to investigate the correlation between the neutrophil-to-lymphocyte ratio (NLR), platelet-to-lymphocyte ratio (PLR), platelet-to-neutrophil ratio (PNR), platelet-to-white blood cell ratio (PWR) and 90-day mortality in patients with acute ischemic stroke (AIS).

**Methods:**

We retrospectively included 633 patients with AIS from January 2017 to May 2018. The correlation between each indicator and the degree of neurologic deficit was assessed. Kaplan-Meier survival curves based on blood cell ratios were used to analyze the 90-day survival rate of patients with AIS.

**Results:**

A total of 663 patients with AIS were enrolled, of which 24 (3.6%) experienced recurrence and 13 (2.0%) died. NLR>3.23 (odds ratio; OR = 2.236; 95% confidence interval [CI], 1.472–3.397; P<0.001), PNR<31.14 (OR = 0.471; 95% CI, 0.297–0.749; P = 0.001), and PWR<20.62 (OR = 0.498; 95% CI, 0.309–0.800; P = 0.004) were associated with an unfavorable 90-day prognosis. NLR>3.23, PWR<20.62, and PNR<31.14 were associated with an increased risk of 90-day mortality.

**Conclusion:**

PNR, PWR, and NLR were associated with the 90-day mortality of patients with AIS. Patients with high NLRs or low PWRs and PNRs may have a greater risk of mortality than other patients. These clinical indicators may help clinicians judge unfavorable prognosis early and implement the appropriate interventions.

## Introduction

Acute ischemic stroke (AIS), a common clinical cerebrovascular disease, has high disability, recurrence and mortality rates in China [1]. The main cause of AIS is atherosclerosis [2], in which inflammation plays an important role [3, 4]. Leukocytes and their subtypes, as commonly used inflammatory markers in the clinic, are considered to be correlated with infarct volume [5], infarct severity [6], and unfavorable outcomes [7]. In addition, many studies have suggested that peripheral blood cell ratios have critical clinical value as novel biomarkers for predicting stroke [8–10].

Previous studies of the relationship between blood cell ratios and stroke mortality had relatively small sample sizes. In addition, studies of the platelet-to-neutrophil ratio (PNR) and platelet-to-white blood cell ratio (PWR) in patients with AIS are limited. Our study retrospectively analyzed 663 patients with AIS to explore the relationship between these novel biomarkers and the mortality of AIS.

## Materials and methods

### Data

Based on a retrospective cohort study, we analyzed 633 patients with AIS admitted to Jiangsu Provincial Hospital of Traditional Chinese Medicine from January 2017 to May 2018.

This study was approved by the scientific research department of the hospital and carried out in accordance with the Declaration of Helsinki and approved by the Institutional Research Review Board at Affiliated Hospital of Nanjing University of Chinese Medicine (2017NL-012-01). Written informed consent was obtained from the patients or their guardians.

### Participants

We included patients with AIS from January 2017 to May 2018. All patients were confirmed by magnetic resonance imaging (MRI) [11]. Patients were excluded if they (1) had bleeding on the first CT examination; (2) had a history of infection within 1 week before symptom onset or were known infections on admission; (3) had a history of malignant tumors; (4) had hematological diseases; (5) could not complete blood cell count within 24 hours of admission; or (6) were aged < 18 years.

### Clinical assessment

The baseline information on admission was collected as follows: age, sex, previous medical history (hypertension, diabetes, hyperlipidemia, atrial fibrillation (AF), coronary heart disease), National Institutes of Health Stroke Scale (NIHSS) score, the Oxfordshire Community Stroke Project (OCSP) classification and treatment after admission (antiplatelet, anticoagulant, lipid-lowering and thrombolysis).

Venous blood samples were obtained within 24 hours after admission. If the blood was examined more than once in 24 hours, we used the first test result for analysis. White blood cell count, neutrophil count, lymphocyte count, and platelet count were determined by Coulter LH750 automatic blood cell analyzer (Beckman, USA). The neutrophil-to-lymphocyte ratio (NLR) was calculated as the absolute value of the ratio of neutrophils to lymphocytes, the platelet-to-lymphocyte ratio (PLR) was calculated as the absolute value of the ratio of platelets to lymphocytes, the PNR was calculated as the absolute value of the ratio of platelets to neutrophils, and the PWR was calculated as the absolute value of the ratio of platelets to white blood cells.

### Follow-up assessments

Ninety days after the onset of AIS, trained graduate students followed up with patients by telephone. Guardians were contacted if patients died or could not cooperate with the inquiry. Patients were evaluated according to the modified Rankin score (mRS) scale. Patients with mRS > 2 were defined as having an unfavorable prognosis, and patients with mRS ≤ 2 were defined as having a favorable prognosis. In addition, stroke recurrence and death in patients were also recorded.

### Statistical analysis

T-tests or Mann-Whitney U tests were used to compare continuous variables between groups according to data distribution; chi-square tests were used to compare the categorical data. The Spearman correlation test was used to analyze the correlation between each indicator and the NIHSS score. The receiver operating characteristic (ROC) curve was used to test the discrimination capability of each indicator for 90-day prognosis and to determine the optimal cutoff value with the highest sum of sensitivity and specificity. According to the optimal cutoff value, the Kaplan-Meier survival curve and log rank tests were performed with each indicator. To avoid missing some important confounding factors, risk factors with a P-value of < 0.1 in the univariate analysis were included in the multivariate analysis. The results were reported as odds ratios (ORs) with 95% confidence intervals (CIs). All statistical analyses were performed using the Statistical Package for the Social Sciences 25.0 (SPSS; IBM, USA), and a two-sided P < 0.05 was considered statistically significant.

## Results

**Fig 1:**
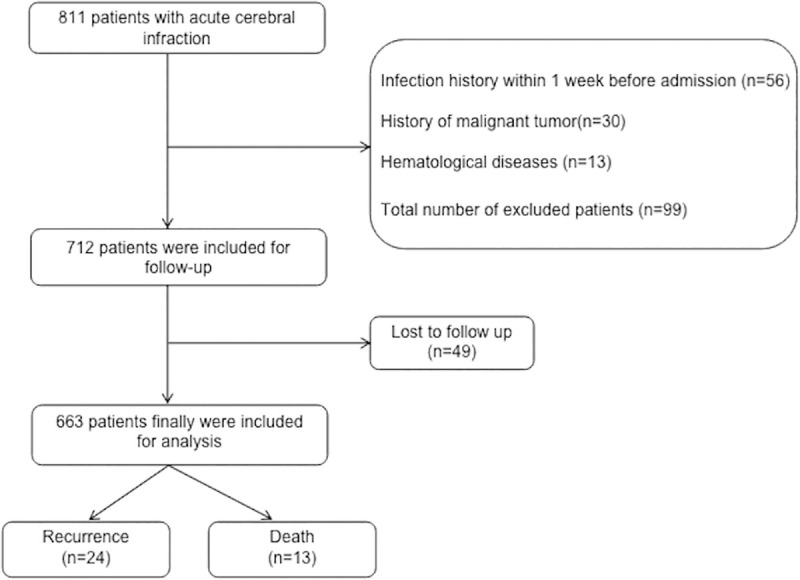
Flow chart of patient enrollment in this study.

A total of eight hundred and eleven patients were admitted to the Encephalopathy Center of Jiangsu Traditional Chinese Medicine Hospital, and one hundred forty-eight patients were excluded from the study. Thirty patients had a history of malignant tumors, fifty-six patients had a history of infections within one week before admission, thirteen patients had a history of hematological diseases, and forty-nine patients were lost to follow-up. Six hundred and sixty-three patients were finally included in the study. A flowchart of patient enrollment is shown in Fig 1.

The demographic, clinical and laboratory characteristics of the patients are shown in Table 1.

**Table 1:** Baseline characteristics of patients with acute ischemic stroke (AIS).

	n = 663
Age, mean (SD)	66.81 (12.58)
Male, n (%)	451 (68.0)
Hypertension	515 (77.7)
Diabetes	262 (39.5)
Dyslipidemia	34 (5.1)
Coronary heart disease	87 (13.1)
AF	56 (8.4)
NIHSS, median (IQR)	3 (2–6)
WBC, median (IQR)	6.80 (5.6–8.0)
Neutrophil, median (IQR)	4.30 (3.5–5.5)
Lymphocyte, median (IQR)	1.60 (1.2–2.0)
NLR, median (IQR)	2.73 (2.1–3.9)
PLR, median (IQR)	114.29 (88.8–157.5)
PNR, median (IQR)	41.75 (32.09–53.59)
PWR, median (IQR)	26.69 (21.30–32.96)
OCSP classification	
TACI	26 (3.9)
PACI	246 (37.1)
POCI	254 (38.3)
LACI	137 (20.7)
Treatment after admission	
Antiplatelet	631 (95.2)
Anticoagulation	281 (42.4)
Thrombolysis	20 (3.0)
Lipid-lowering	646 (97.4)

Abbreviations: AF, Atrial Fibrillation; NIHSS, National Institutes of Health Stroke Scale; WBC, White Blood Cell; NLR, Neutrophil-to-Lymphocyte Ratio; PLR, Platelet-to-Lymphocyte Ratio; PNR, Platelet-to-Neutrophil Ratio; PWR, Platelet-to-White Blood Cell Ratio; OCSP, Oxfordshire Community Stroke Project; TACI, Total Anterior Cerebral Infarction; PACI, Partial Anterior Cerebral Infarction; POCI, Posterior Cerebral Infarction; LACI, Lacunar Cerebral Infarction.

Four hundred fifty-one (68%) patients were males, with an average age of 66.81±12.58 (range 24 to 99 years old). Five hundred and fifteen (77.7%) patients had hypertension, two hundred and sixty-two (39.5%) patients had diabetes, thirty-four (5.1%) patients had dyslipidemia, eighty-seven (13.1%) patients had coronary heart disease, and fifty-six (8.4%) patients had AF. The general treatment during hospitalization for patients included in this study was antiplatelet (95.2%) or anticoagulant (42.4%) therapy; twenty (3.0%) patients received thrombolytic therapy, and six hundred forty-six (97.4%) patients were treated with statins.

As shown in Table 2, the NIHSS scores were positively correlated with NLR (rho = 0.291, P < 0.001) and PLR (rho = 0.171, P < 0.001) and inversely correlated with PNR (rho = -0.185, P < 0.001) and PWR (rho = -0.098, P = 0.012), among which NLR had the closest correlation.

**Table 2:** The correlation between National Institutes of Health Stroke Scale (NIHSS) scores on admission and each indicator.

	rho	*P-*value
NLR	0.291	<0.001
PLR	0.171	<0.001
PNR	-0.185	<0.001
PWR	-0.098	0.012

Abbreviations: NIHSS, National Institutes of Health Stroke Scale; NLR, Neutrophil-to-Lymphocyte Ratio; PLR, Platelet-to-Lymphocyte Ratio; PNR, Platelet-to-Neutrophil Ratio; PWR, Platelet-to-White Blood Cell Ratio.

Furthermore, according to the mRS score at 90 days of follow-up, the patients were divided into favorable prognosis (mRS ≤ 2) and unfavorable prognosis (mRS > 2). As shown in Table 3, in the whole group, four hundred and eighty-seven (73.5%) patients had a favorable prognosis, and one hundred and seventy-six (26.5%) patients had an unfavorable prognosis.

**Table 3:** Baseline characteristics for patients with different prognoses.

Characteristics	Unfavorable	Favorable	*P-*value
	n = 176	n = 487	
Age, mean (SD)	71.59 (11.87)	65.08 (12.39)	0.899
Male, n (%)	109 (61.9)	342 (70.2)	0.043
Hypertension, n (%)	142 (80.7)	373 (76.6)	0.264
Diabetes, n (%)	86 (48.9)	176 (36.1)	0.003
Dyslipidemia, n (%)	5 (2.8)	29 (6.0)	0.108
Coronary heart disease, n (%)	36 (20.5)	51 (10.5)	0.001
AF, n (%)	29 (16.5)	27 (5.5)	<0.001
NIHSS, median (IQR)	6 (4–11)	3 (1–4)	<0.001
WBC, median (IQR)	7.23 (6.2–8.5)	6.60 (5.5–7.8)	<0.001
Neutrophil, median (IQR)	5.12 (3.9–6.3)	4.20 (3.3–5.2)	<0.001
Lymphocyte, median (IQR)	1.40 (1.0–1.8)	1.60 (1.3–2.1)	<0.001
NLR, median (IQR)	3.47 (2.5–5.7)	2.57 (1.9–3.5)	<0.001
PLR, median (IQR)	125.42 (94.5–190.3)	110.91 (87.5–145.6)	<0.001
PNR, median (IQR)	37.13 (27.81–48.49)	43.25 (34.41–54.81)	<0.001
PWR, median (IQR)	25.56 (19.50–41.64)	27.17 (22.22–33.33)	0.002
OCSP classification			<0.001
TACI	14 (8.0)	12 (2.5)	
PACI	66 (37.5)	180 (37.0)	
POCI	71 (40.3)	183 (37.6)	
LACI	25 (14.2)	112 (223.0)	
Treatment after admission			
Antiplatelet	164 (93.2)	467 (95.9)	0.150
Anticoagulation	93 (52.8)	188 (38.6)	0.001
Thrombolysis	6 (3.4)	14 (2.9)	0.722
Lipid-lowering	167 (94.9)	479 (98.4)	0.022

Abbreviations: AF, Atrial Fibrillation; NIHSS, National Institutes of Health Stroke Scale; WBC, White Blood Cell; NLR, Neutrophil-to-Lymphocyte Ratio; PLR, Platelet-to-Lymphocyte Ratio; PNR, Platelet-to-Neutrophil Ratio; PWR, Platelet-to-White Blood Cell Ratio; OCSP, Oxfordshire Community Stroke Project; TACI, Total Anterior Cerebral Infarction; PACI, Partial Anterior Cerebral Infarction; POCI, Posterior Cerebral Infarction; LACI, Lacunar Cerebral Infarction.

**Fig 2:**
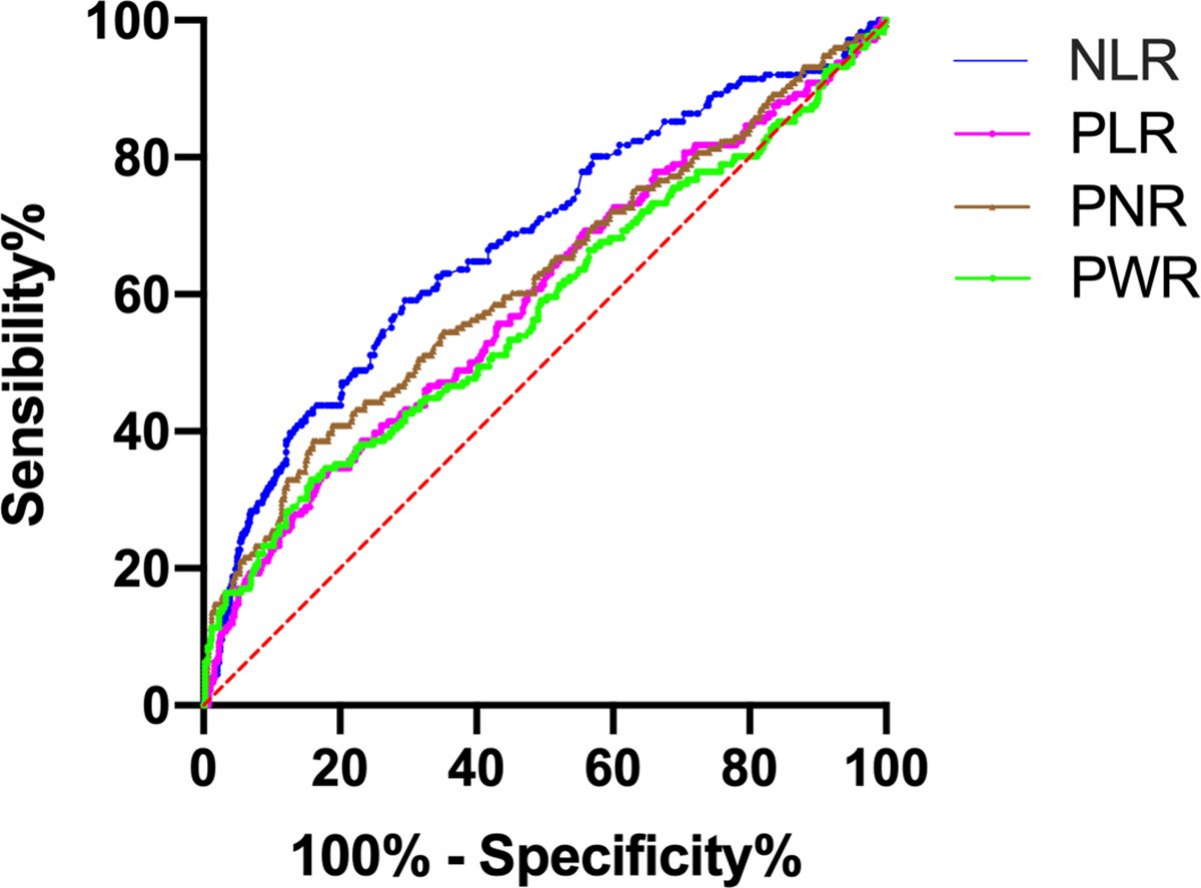
Receiver Operating Characteristic (ROC) curve of each indicator on the prognosis of patients with AIS.

Patients with an unfavorable prognosis were older with a smaller proportion of males, higher initial NIHSS score, and higher rates of hypertension, diabetes, AF, and coronary heart disease than those with a favorable prognosis. The ROC curves of the predictive value of each indicator for unfavorable prognosis are shown in Fig 2.

The area under the ROC curve (AUC) of the NLR was 0.678, the Youden index was 0.295, the optimal cutoff value was 3.23, the sensitivity was 59.1%, and the specificity was 70.4%. The AUC of PLR was 0.593, the Youden index was 0.164, the optimal cutoff value was 163.12, the sensitivity was 34.7%, and the specificity was 81.7%. The AUC of PNR was 0.620, the Youden index was 0.226, the optimal cutoff value was 31.14, the sensitivity was 38.6%, and the specificity was 84.0%. The AUC of PWR was 0.577, the Youden index was 0.172, the optimal cutoff value was 20.62, the sensitivity was 33.0%, and the specificity was 84.2%.

In addition, logistic regression analysis was performed with each indicator, which was included as binary variables dichotomized according to the optimal cutoff value. Table 4 summarizes the results of the multivariate analysis.

**Table 4:** Multivariate logistic analysis of each indicator for 90-day prognosis.

	Adjusted OR (95% CI)	P-value
NLR (>3.23 vs ≤3.23)	2.236 (1.472–3.397)	<0.001
PLR (≥163.12 vs <163.12)	1.565 (0.984–2.489)	0.058
PNR (≥31.14 vs <31.14)	0.471 (0.297–0.749)	0.001
PWR (≥20.62 vs <20.62)	0.498 (0.309–0.800)	0.004

Abbreviations: NLR, Neutrophil-to-Lymphocyte Ratio; PLR, platelet-to-lymphocyte ratio; PNR, platelet-to-neutrophil ratio; PWR, platelet-to-white blood cell ratio.

*Adjustment for sex, diabetes, atrial fibrillation, coronary heart disease, NIHSS score, OCSP classification, anticoagulant, lipid-lowering.

**Fig 3:**
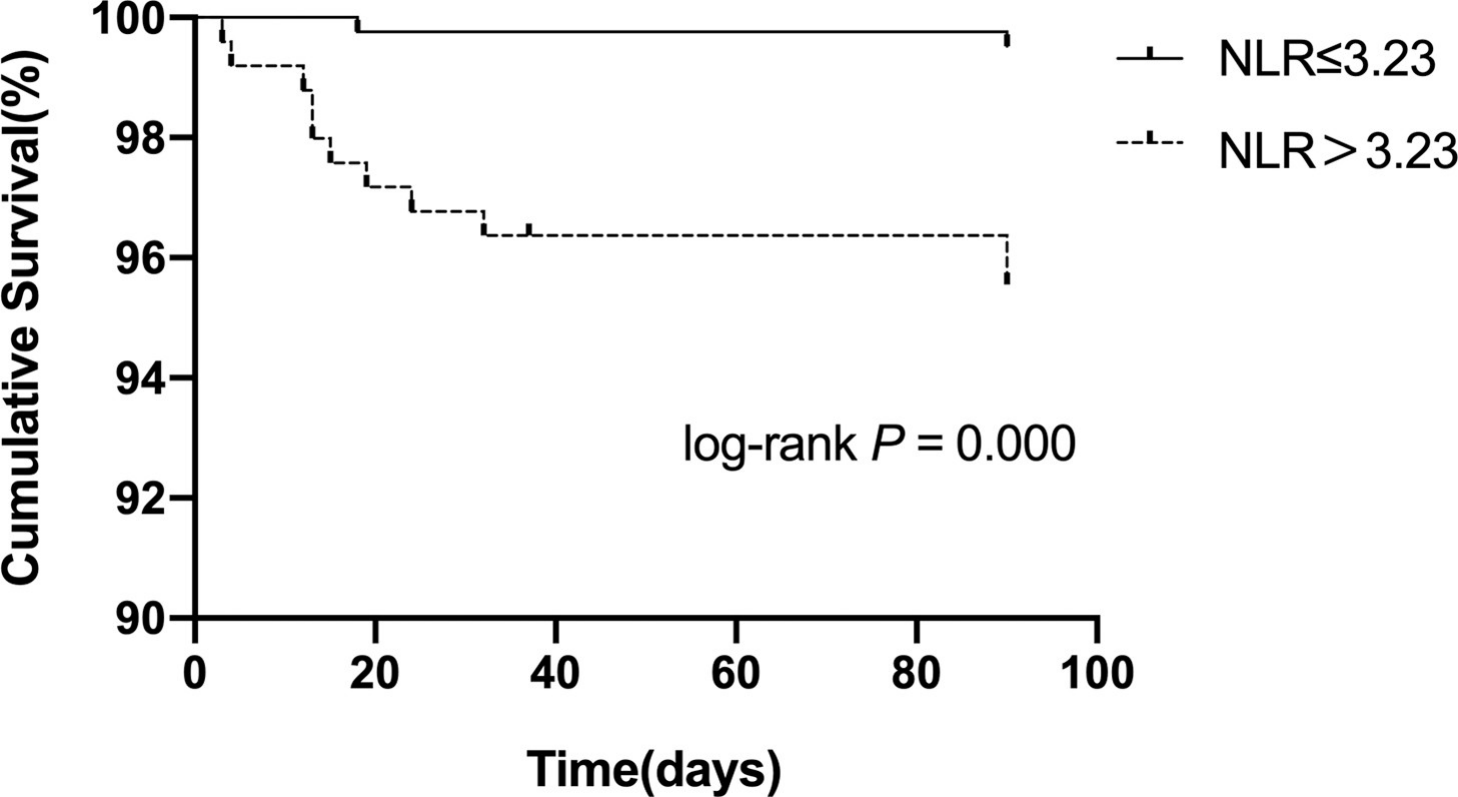
Kaplan-Meier survival curve for 90-day mortality in patients with AIS according to the NLR.

**Fig 4:**
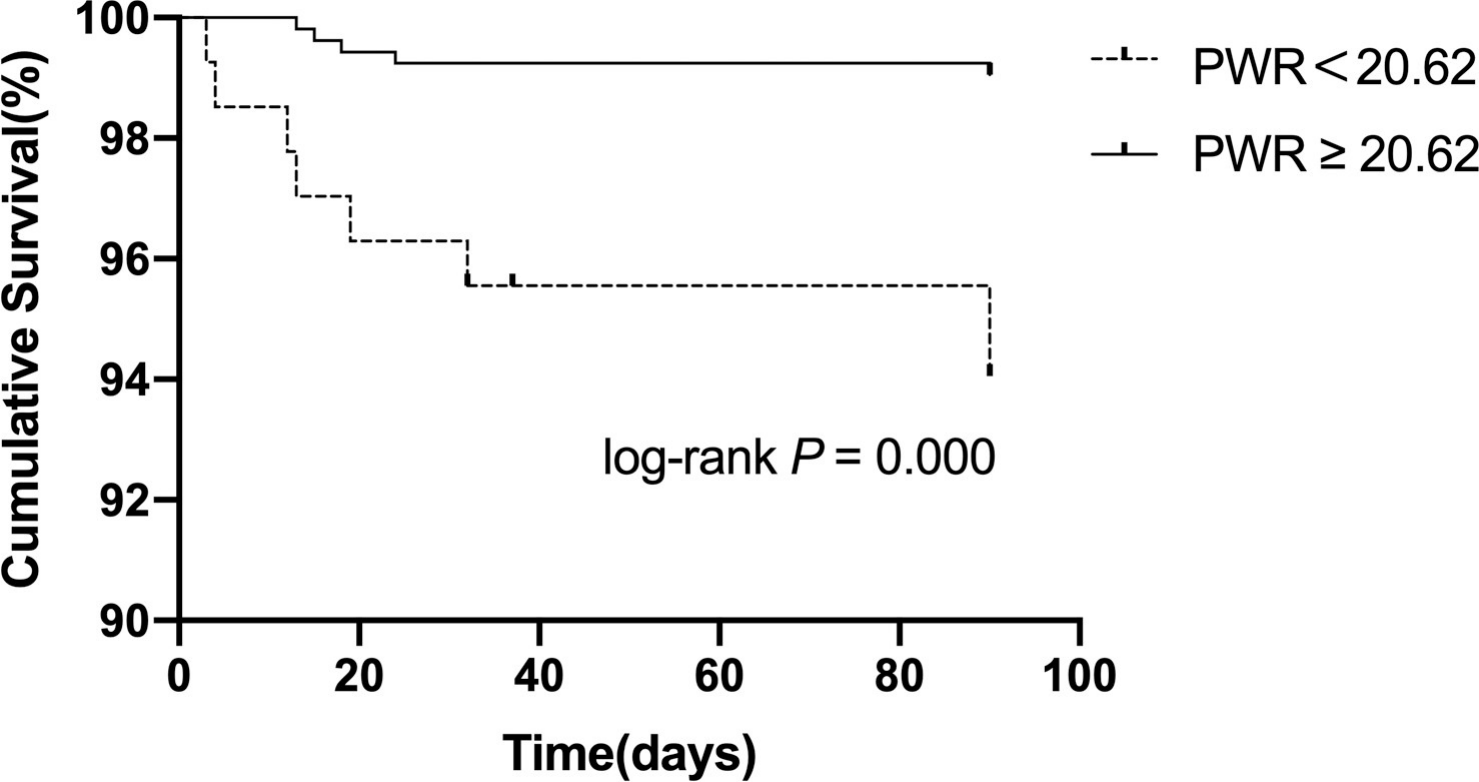
Kaplan-Meier survival curve for 90-day mortality in patients with AIS according to the PWR.

**Fig 5:**
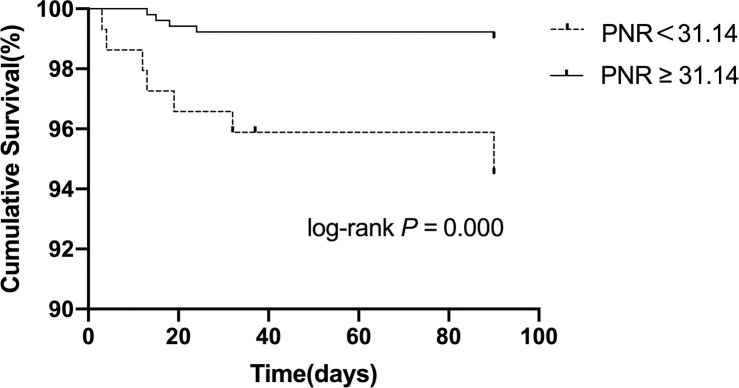
Kaplan-Meier survival curve for 90-day mortality in patients with AIS according to the PNR.

After adjusting for sex, diabetes, coronary heart disease, AF, NIHSS score, OCSP classification, anticoagulation and lipid-lowering, NLR (OR = 2.236;95% CI 95% CI, 1.472–3.397; *P* < 0.001), platelet-to-neutrophil ratio<31.14 (OR = 0.471; 95% CI, 0.297–0.749; P = 0.001), and platelet-to-white blood cell ratio<20.62 (OR = 0.498; 95% CI, 0.309–0.800; P = 0.004) were found to be independently correlated with unfavorable functional prognosis at 90 days. Moreover, we further explored whether each indicator was associated with 90-day stroke recurrence or mortality. As shown in Figs 3–5, and Table 5, the total recurrence rate was 3.6% (24/663), and the mortality rate was 2.0% (13/663) at 90 days. NLR>3.23, PWR<20.62, and PNR<31.14 were associated with a low risk of 90-day mortality.

**Table 5:** The 90-day stroke recurrence and mortality of AIS patients.

	Total	Recurrence	Log rank *P*	Mortality	Log rank *P*
NLR					
>3.23	248	10 (4.0%)	0.589	11 (4.4%)	<0.001
≤3.23	415	14 (3.4%)		2 (0.5%)	
PLR					
≥163.12	150	6 (4.0%)	0.747	4 (2.7%)	0.478
<163.12	513	18 (3.5%)		9 (1.8%)	
PNR					
≥31.14	517	20 (3.9%)	0.565	5 (1.0%)	<0.001
<31.14	146	4 (2.7%)		8 (5.5%)	
PWR					
≥20.62	528	20 (3.8%)	0.711	5 (0.9%)	<0.001
<20.62	135	4 (3.0%)		8 (5.9%)	

Abbreviations: AIS, Acute ischemic stroke; NLR, Neutrophil-to-Lymphocyte Ratio; PLR, platelet-to-lymphocyte ratio; PNR, platelet-to-neutrophil ratio; PWR, platelet-to-white blood cell ratio.

## Discussion

In our study, we found that the severity of acute ischemic stroke (AIS) was positively correlated with the neutrophil-to-lymphocyte ratio (NLR) and platelet-to-lymphocyte ratio (PLR) and inversely correlated with the platelet-to-neutrophil ratio (PNR) and platelet-to-white blood cell ratio (PWR). An elevated NLR was independently associated with an unfavorable 90-day functional prognosis of AIS. In addition, a decreased PNR and PWR also indicated a poor functional prognosis in patients. Additionally, the NLR, PNR, and PWR were linked to the 90-day mortality of patients with AIS. Lower levels of PNR and PWR were associated with a greater risk of death, which indicated that PNR and PWR may be autocephaly protective predictors of the 90-day mortality of AIS patients. In addition, considering the low proportion of patients with thrombolytic treatment and lacunar stroke, it seemed that the mortality (2.0%) in our study was relatively low, which may be due to the relatively short follow-up time. Additionally, the application of Argatroban injection [12] and Chinese patent medicine injection (such as Diterpene ginkgolides meglumine injection [13] and gastrodin injection [14]) may contribute to ischemic injury and the short-term prognosis of patients.

The relationship between ischemic stroke and peripheral leukocytes is complex and bidirectional. Ischemic stroke can cause immune disorders and induce systemic inflammatory reactions characterized by changes in peripheral leukocytes [15]. In turn, activated leukocytes can aggravate neuronal damage and expand infarct sizes through various pathophysiological pathways [16, 17]. Previous studies [18, 19] proved that there was a significant correlation between stroke severity and peripheral leukocyte count. An experimental study [20] using the intraluminal middle cerebral artery occlusion (MCAO) mouse model of ischemic stroke showed that a larger infarction volume was associated with more prolonged and pronounced changes in immune cells (such as neutrophils and lymphocytes) after stroke.

White blood cells and their subtypes have different prognostic roles in AIS. Studies have shown that elevated white blood cell counts increase the risk of cerebral infarction and are associated with poor outcomes in patients with AIS [21, 22]. Neutrophils are peripheral immune cells that first infiltrate the ischemic area. Activated neutrophils secrete some harmful substances and inflammatory mediators (e.g., cellular adhesion molecules, reactive oxygen species, and matrix metalloproteinases) that can exacerbate ischemic injury [23] and even induce hemorrhagic transformation [24]. Additionally, neutrophil extracellular traps (NETs) released by neutrophils are considered to be another potential mechanism leading to blood-brain barrier damage and hemorrhagic transformation, which may be the reason why neutrophils are associated with poor outcomes [25]. The role of lymphocytes in AIS is controversial. Studies confirmed that lymphocyte counts significantly decreased after stroke onset [26, 27]. Activated lymphocytes can aggravate brain tissue damage by releasing NADPH oxidase [28], which has negative impacts on neuroprotection. Therefore, the reduction in lymphocytes is considered to be an internal self-protection mechanism.

Many studies have shown that the NLR is closely related to poor functional outcomes and short-term mortality of AIS [29, 30]. This is consistent with our results. High neutrophil counts worsen the inflammatory reaction in ischemic areas, exacerbating brain edema and neuron death. Low lymphocyte counts maintained the body's immune response to ischemic areas. Thus, an elevated NLR suggests stronger inhibition of the inflammatory reaction and immune response.

Platelets are activated after being stimulated by various factors, including inflammation and atherosclerosis. Activated platelets accumulate at sites of damaged endothelial cells, releasing proinflammatory mediators. Furthermore, activated platelets are involved in the development of atherosclerosis, which will gradually lead to the rupture of atherosclerotic plaques and trigger ischemic events [31, 32]. Many studies [23, 33] have elaborated that activated platelets release chemicals related to leukocyte recruitment and interact with white blood cells and neutrophils [34], which exacerbate inflammation and thrombosis. Moreover, the reason for the PWR or PNR to predict death in AIS patients may be that the platelet-leukocyte complex exacerbates ischemia-reperfusion injury [35]. When AIS occurs, thrombosis causes excessive depletion of platelets, leading to a decrease in platelet counts [36, 37]. Therefore, PWR and PNR can fully reflect the degree of thrombosis and inflammation.

The PLR was demonstrated to be an independent predictor of diabetes with chronic complications [38–40] and tumors (such as non-small-cell lung cancer and hypopharyngeal squamous cell carcinoma) [41, 42]. Recent studies proved that PLR is related to the prognosis of stroke thrombolysis [43] or endovascular treatment [44]. However, we did not find a significant correlation between PLR and unfavorable functional prognosis or adverse events in AIS patients. The possible reason may be that there were differences in the blood sample collection time and heterogeneity of patients.

There are some limitations to our study. First, the patients included in this study were all from one center and do not represent all Chinese people. Thus, more multicenter and prospective studies are needed. Second, this is a hospital-based study that could have potential selection bias, and possible confounders cannot be completely excluded. Finally, as this is a retrospective study, we could not measure all the indicators continuously to observe the dynamic changes in each indicator.

## Conclusion

PNR, PWR, and NLR were associated with the 90-day mortality of patients with AIS. Patients with high NLRs or low PWRs and PNRs may suffer a greater risk of mortality than other patients. These convenient and easily obtained indicators may help clinicians judge prognosis early and implement the appropriate interventions.

## Supporting information

S1 Checklist Clinical studies checklist. (DOCX)

S2 Checklist STROBE checklist. (DOCX)

S1 Data Relevant data underlying the findings described in manuscript. (XLSX)

S1 File Jiangsu province administration of Chinese medicine-1. (PDF)

S2 File 333 High level talents training project in Jiangsu-2. (PDF)
