# Supplementary material for: The correlation between novel peripheral blood cell ratios and 90-day mortality in patients with acute ischemic stroke
Source: PLoS One. 2020 Aug 28;15(8):e0238312. doi: 10.1371/journal.pone.0238312 (PMC7454963; doi:10.1371/journal.pone.0238312)
Supplement: S1 File — (PDF) [file pone.0238312.s004.pdf]

伦理审查批件

<table>
<tr><td>批件号</td><td colspan="3">2017NL-012-01</td></tr>
<tr><td>项目名称</td><td colspan="3">基于“瘀热阻窍”治疗急性脑梗死的队列研究</td></tr>
<tr><td>项目来源</td><td colspan="3">江苏省中医药局科研专项课题</td></tr>
<tr><td>研究单位</td><td colspan="3">江苏省中医院</td></tr>
<tr><td>主要研究者</td><td colspan="3">吴明华</td></tr>
<tr><td>审查类别</td><td>初始审查申请</td><td>审查方式</td><td>会议审查</td></tr>
<tr><td>审查日期</td><td>2017 年 03 月 29 日</td><td>审查地点</td><td>江苏省中医院 5 号楼 402 室</td></tr>
<tr><td>审查委员</td><td colspan="3">吴明华、刘兰英、刘鸣、邱召娟、曲宁、王昕、徐玉红、徐中芹、姚昶、朱磊、朱永康</td></tr>
<tr><td>审查批准文件</td><td colspan="3">临床研究方案 版本号：2.0 版本日期：2016-10-12
知情同意书 版本号：1.0 版本日期：2017-03-16</td></tr>
<tr><td colspan="4">审查意见</td></tr>
<tr><td colspan="4">根据国家卫生计生委《涉及人的生物医学研究伦理审查办法》（2016）、CFDA《药物临床试验质量管理规范》（2003）、《医疗器械临床试验质量管理规范》（2016）、WMA《赫尔辛基宣言》和 CIOMS《人体生物医学研究国际道德指南》的伦理原则，经本伦理委员会审查，同意按所批准的临床研究方案、知情同意书、招募材料开展本项研究。
请遵循 GCP 原则、遵循伦理委员会批准的方案开展临床研究，保护受试者的健康与权利。
经伦理委员会批准的研究项目在实施前，研究项目负责人应当将该研究项目的主要内容、伦理审查决定在医学研究登记备案信息系统进行登记。凡涉及中国人类遗传资源、需要报批的研究项目，应在获得中国人类遗传资源管理办公室批准后才能开始研究。
研究过程中若变更主要研究者，对临床研究方案、知情同意书、招募材料等的任何修改，请申请人提交修正案审查申请。
发生严重不良事件，请申请人及时提交严重不良事件报告。
请按照伦理委员会规定的年度/定期审查频率，申请人在截止日期前 1 个月提交研究进展报告；申办者应当向组长单位伦理委员会提交各中心研究进展的汇总报告；当出现任何可能显著影响试验进行、或增加受试者危险的情况时，请申请人及时向伦理委员会提交书面报告。超出批件有效期，没有提交研究进展报告并获得伦理审查批准继续研究的项目，研究者必须立即停止所有研究活动，包括干预措施和数据收集。假若停止研究干预可能会对受试者造成伤害，研究者应当要求伦理委员会批准在研的受试者继续参加研究。
研究纳入了不符合纳入标准或符合排除标准的受试者，符合中止试验规定而未让受试者退出研究，给予错误治疗或剂量，给予方案禁止的合并用药等没有遵从方案开展研究的情况；或可能对受试者的权益/健康、以及研究的科学性造成不良影响等违背 GCP 原则的情况，请申办者/监查员/研究者提交违背方案报告。
申请人暂停或提前终止临床研究，请及时提交暂停/终止研究报告。
完成临床研究，请申请人提交研究完成报告，以及概述研究发现和结论的总结报告。</td></tr>
<tr><td>年度/定期跟踪审查频率</td><td colspan="3">请于 2018 年 03 月 29 日前 1 个月提交研究进展报告</td></tr>
<tr><td>有效期</td><td colspan="3">12 个月</td></tr>
<tr><td>联系人与联系电话</td><td colspan="3">吴静 025-86560515</td></tr>
<tr><td>主席签字</td><td colspan="3">[signature]</td></tr>
<tr><td>伦理委员会</td><td colspan="3">南京中医药大学附属医院（江苏省中医院）伦理委员会（盖章）</td></tr>
<tr><td>日期</td><td colspan="3">2017 年 03 月 30 日</td></tr>
</table>

南京中医药大学附属医院伦理委员会
