# Supplementary material for: The correlation between novel peripheral blood cell ratios and 90-day mortality in patients with acute ischemic stroke
Source: PLoS One. 2020 Aug 28;15(8):e0238312. doi: 10.1371/journal.pone.0238312 (PMC7454963; doi:10.1371/journal.pone.0238312)
Supplement: S2 File — (PDF) [file pone.0238312.s005.pdf]

伦理审查批件

批件号	2018NL-033-02		
项目名称	基于“瘀热阻窍”和CYP2C19基因突变治疗急性脑梗死颅内动脉狭窄的研究		
项目来源	2016年度省第五期“333工程”科研项目		
研究单位	江苏省中医院		
主要研究者	吴明华		
审查类别	复审申请	审查方式	快速审查
审查日期	2018年05月10日	审查地点	
审查委员	刘鸣		
审查批准文件	修正的临床研究方案 版本号：2.0 版本日期：2018-05-09<br>修正的知情同意书 版本号：2.0 版本日期：2018-05-09		

审查意见

根据国家卫生计生委《涉及人的生物医学研究伦理审查办法》(2016)、CFDA《药物临床试验质量管理规范》(2003)、《医疗器械临床试验质量管理规范》(2016)、WMA《赫尔辛基宣言》和 CIOMS《涉及人的生物医学研究国际伦理审查指南》的伦理原则，经本伦理委员会审查，同意按所批准的临床研究方案、知情同意书、招募材料开展本项研究。

请遵循 GCP 原则、遵循伦理委员会批准的方案开展临床研究，保护受试者的健康与权利。

经伦理委员会批准的研究项目在实施前，研究项目负责人应当将该研究项目的主要内容、伦理审查决定在医学研究登记备案信息系统进行登记。凡涉及中国人类遗传资源、需要报批的研究项目，应在获得中国人类遗传资源管理办公室批准后才能开始研究。

研究过程中若变更主要研究者，对临床研究方案、知情同意书、招募材料等的任何修改，请申请人提交修正案审查申请。

发生严重不良事件，请申请人及时提交严重不良事件报告。

请按照伦理委员会规定的年度/定期审查频率，申请人在截止日期前 1 个月提交研究进展报告；申办者应当向组长单位伦理委员会提交各中心研究进展的汇总报告；当出现任何可能显著影响试验进行、或增加受试者危险的情况时，请申请人及时向伦理委员会提交书面报告。超出批件有效期，没有提交研究进展报告并获得伦理审查批准继续研究的项目，研究者必须立即停止所有研究活动，包括干预措施和数据收集。假若停止研究干预可能会对受试者造成伤害，研究者应当要求伦理委员会批准在研的受试者继续参加研究。

研究纳入了不符合纳入标准或符合排除标准的受试者，符合中止试验规定而未让受试者退出研究，给予错误治疗或剂量，给予方案禁止的合并用药等没有遵从方案开展研究的情况；或可能对受试者的权益/健康、以及研究的科学性造成不良影响等违背 GCP 原则的情况，请申办者/监查员/研究者提交违背方案报告。

申请人暂停或提前终止临床研究，请及时提交暂停/终止研究报告。

完成临床研究，请申请人提交研究完成报告，以及概述研究发现和结论的总结报告。

年度/定期跟踪审查频率	请于2019年05月10日前1个月提交研究进展报告
有效期	12个月
联系人与联系电话	吴静 025-86560515
主席签字	刘鸣
伦理委员会	南京中医药大学附属医院（江苏省中医院）伦理委员会（盖章）
日期	2018年05月10日
